# Fragility Limits Performance in Complex Networks

**DOI:** 10.1038/s41598-020-58440-6

**Published:** 2020-02-04

**Authors:** Fabio Pasqualetti, Shiyu Zhao, Chiara Favaretto, Sandro Zampieri

**Affiliations:** 1 University of California at Riverside, Department of Mechanical Engineering, Riverside, 92521 CA USA; 2 Westlake University, Shcool of Engineering, Hangzhou, 310024 China; 3 University of Padova, Department of Information Engineering, Padova, 35131 Italy; 4 University of Padova, Department of Neuroscience, Padova, 35128 Italy

**Keywords:** Electrical and electronic engineering, Applied mathematics

## Abstract

While numerous studies have suggested that large natural, biological, social, and technological networks are fragile, convincing theories are still lacking to explain why natural evolution and human design have failed to optimize networks and avoid fragility. In this paper we provide analytical and numerical evidence that a tradeoff exists in networks with linear dynamics, according to which general measures of robustness and performance are in fact competitive features that cannot be simultaneously optimized. Our findings show that large networks can either be robust to variations of their weights and parameters, or efficient in responding to external stimuli, processing noise, or transmitting information across long distances. As illustrated in our numerical studies, this performance tradeoff seems agnostic to the specific application domain, and in fact it applies to simplified models of ecological, neuronal, and traffic networks.

## Introduction

**Figure 1:**
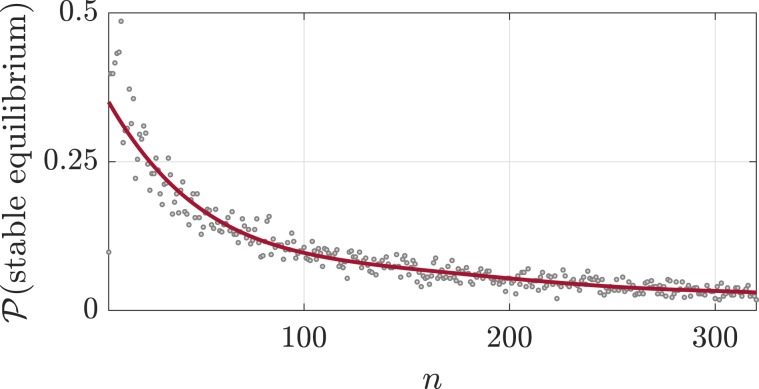
This figure shows the probability that an ecological network is stable as a function of the network dimension. Each point in the plot represents the percentage of stable networks with given dimension (*n*), over a sample of 500 mutualistic ecological networks^47^ with randomly chosen small-world topology. As can be seen, the percentage of stable networks decreases to zero as the network cardinality increases. This suggests that ecological networks with increasing size are likely unstable or fragile, so that small parameter variations lead to unstable evolutions.

Across diverse scientific disciplines and application domains, complex systems are commonly represented as the dynamic interconnection of nodes and edges (namely, networks), where the interaction pattern among different parts is itself complex and may evolve along with the system dynamics. With this formalism, nodes and edges correspond, for instance, to populations of neurons and their functional relations in neuronal networks^1^, to different species and their trophic interactions in ecological networks^2^, or to generators, loads and connection lines in power networks^3^. Node sets are typically large; interconnections sparse and heterogeneous. Despite being able to accomplish a rich set of dynamic functions through different nodal and interconnection dynamics, many complex networks exhibit fragile behaviors against relatively small variations of the edge weights and interconnection structure. This is the case in ecological systems, where fragility affects the chance that species can coexist at a stable equilibrium^4^ (see also Fig. 1). In neuronal networks fragility implies, for instance, that small variations in certain synaptic weights can suddenly induce unstable behaviors and cause seizures^5^. Small changes in the interaction weights are also thought responsible for increases in ocean acidity^6^, cascading failures in power systems^7^, and traffic congestions^8^.

Fragility of complex networks stands out as a negative feature that, surprisingly, neither natural evolution nor careful human design have been able to remedy. Existing theories fall short in explaining this phenomenon. For instance, research in network science and graph optimization focuses primarily on static network models and diagnostics^9–13^, which cannot fully explain dynamic network properties. Only fewer and recent papers address dynamic features of networks, such as stability, fragility, and controllability^14–18^, yet fail to create a provable link among these properties and highlight possible causes of fragility. In this article we leverage network- and control-theoretic tools to propose a mechanistic explanation of why several natural and man-made complex systems are fragile. For networks with linear dynamics, we show that (i) a tradeoff exists between the fragility of a network, as measured by its ability to maintain a stable behavior after a perturbation of its edge weights and interconnection structure, and its performance, as measured by its responsiveness to internal and external stimuli, and that (ii) certain network systems may be tuned to sacrifice their robustness in favor of increased responsiveness and adaptability.

## Results

**Figure 2:**
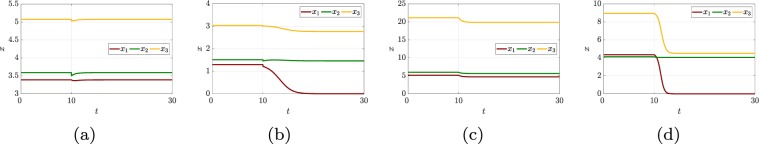
This Figure shows the state evolution in four examples of nonlinear ecological systems with mutualistic interactions^47^ of dimension $n=3$. Panels (a and b) highlight the differences between the dynamics of stable and of unstable equilibria. In panel (a) the three state evolutions are at equilibrium until time $t=10$, after which they are slightly perturbed. Because this equilibrium configuration is stable, the state returns to the equilibrium configuration after a small transient. In panel (b), instead, a small perturbation of the state generates a large deviation from the equilibrium, which drives the system towards a different configuration where one of the three species is absent. Panels (c and d) highlight the difference between a robust and a fragile system. Panel (c) shows the evolution of a robust system: the change of parameters modifies the equilibrium but not its stability properties, allowing the state to converge to a new equilibrium. Panel (d) shows the evolution of a fragile system, where the parameters variation modifies the equilibrium point and its stability properties, rendering the trajectories unstable and leading to the extinction of one specie.

Optimized networks should feature low fragility and high responsiveness, so as to remain stable against accidental parameter variations and yet allow for efficient manipulation from legitimate controls. Unfortunately, this work shows that such networks cannot exist, because fragility and responsiveness are found to be competitive features. To show this, we focus on networks with linear dynamics and adopt a metric of fragility that measures the ability of the network to maintain a stable behavior in the face of changes of the edge weights. In particular, our notion of network fragility, which is borrowed from early and ongoing work in control theory^19,20^, measures the size of the smallest variation of the edge weights leading to dynamic instability – see below and our Supplementary Information for a formal definition of network fragility. In a stable network, small perturbations of the equilibrium have limited network-wide effects and are bound to vanish over time (see Fig. 2(a)). Conversely, unstable networks deviate arbitrarily far from the equilibrium when subject to small perturbations (see Fig. 2(b)).

Dynamic responsiveness represents the ability of a network to change its configuration in response of external stimuli carrying information signals, control actions, or random fluctuations. To quantify the responsiveness of a system we use the control-theoretic notion of controllability Gramian^21^. The controllability Gramian describes how signals propagate across a network, and its eigenvalues can be used to quantify different dynamic properties, including controllability, responsiveness, and excitability. As explained below and in our Supplementary Information, our definition of responsiveness is in fact inspired by a graded notion of network controllability^15^, a metric that has received renewed interest for the study of network systems^22–24^.

We next formalize our definitions, provide a first characterization of the relationships between fragility and responsiveness in networks, and ultimately discuss why fragility and responsiveness cannot be optimized simultaneously, so that fragility of existing networks can be viewed as the byproduct of an optimization process aimed at improving dynamic responsiveness.

### Network model and performance metrics

We study complex systems with linear dynamics arising from the interconnection of heterogeneous units, which are conveniently represented as networks. Formally, a network is described by a directed graph ${\mathscr{G}}=({\mathscr{V}},{\mathscr{E}})$, where ${\mathscr{V}}=\{1,\ldots ,n\}$ and ${\mathscr{E}}\subseteq {\mathscr{V}}\times {\mathscr{V}}$ are the vertex and edge sets, respectively. We assume that a subset of ${n}_{{\rm{c}}}$ nodes, called drivers, can be controlled independently and, to simplify the notation and without affecting generality, we let the drivers be the first ${n}_{{\rm{c}}}$ nodes. Let $A=[{a}_{ij}]$ be the weighted adjacency matrix of ${\mathscr{G}}$, and let ${x}_{i}(t)$ be the time-dependent variable representing the state of node *i*. The dynamics of the nodes are described by the differential equations

$$\begin{array}{llll}\frac{d}{dt}{x}_{i}(t) & = & \mathop{\sum }\limits_{j=1}^{n}\,{a}_{ij}{x}_{j}(t)+{u}_{i}(t), & \,({\rm{driver}}\,{\rm{node}})\\ \frac{d}{dt}{x}_{i}(t) & = & \mathop{\sum }\limits_{j=1}^{n}\,{a}_{ij}{x}_{j}(t), & \,({\rm{non}} \mbox{-} {\rm{driver}}\,{\rm{node}})\end{array}$$

where ${u}_{i}(t)$ is the external input influencing the *i*-th driver. By collecting the states and the inputs into vectors, the network dynamics read as

1 $$\frac{d}{dt}x(t)=Ax(t)+Bu(t),$$

where $B={[I0]}^{{\rm{\top }}}$ is the input matrix and *I* is the identity matrix. This network model, which will also be referred to as linear dynamic network, has been widely adopted in numerous fields to study both natural and man-made complex systems, especially when the dynamics are constrained or informed by an interconnection structure^16,25^.

We will use the notion of stability radius to measure the fragility of the linear dynamic network (1):

2 $$r(A)=\{\parallel \Delta \parallel \,:\,\Delta \in {{\mathbb{C}}}^{n\times n}\,{\rm{and}}\,A+\Delta \,{\rm{is}}\,{\rm{unstable}}\},$$

where ||·|| denotes the Euclidean norm, and $A+\Delta$ is unstable when at least one of its eigenvalues has a positive real part. Loosely speaking, the index $r(A)$ quantifies the minimum size of an edge perturbation that renders the network unstable; thus, the smaller $r(A)$ the more fragile the network to edge perturbations. Further, we use the following metric based on the controllability Gramian to quantify the responsiveness of the linear dynamic network (1):

3 $$\bar{\sigma }\left(G\right)=\bar{\sigma }\left({\int }_{0}^{\infty }\,{e}^{At}B{B}^{\top }{e}^{{A}^{\top }t}dt\right),$$

where $\bar{\sigma }(\,\cdot \,)$ denotes the average singular value of a matrix. We remark that $\bar{\sigma }(G)$ is a direct measure of how the linear dynamic network (1) responds to external inputs, and an indirect measure of the controllability degree of (1). We refer the reader to the section Methods below and the Supplementary Information for a detailed discussion of $r(A)$ and $\bar{\sigma }(G)$.

### Tradeoff between fragility and responsiveness

**Figure 3:**
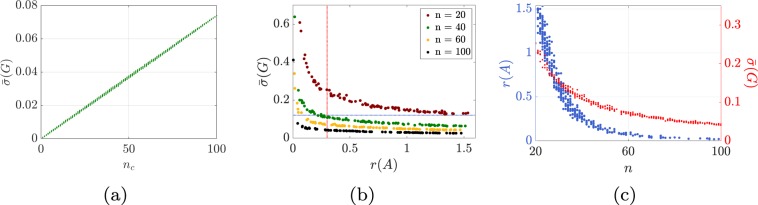
This figure illustrates the tradeoff between fragility and responsiveness described in (4) in synthetic networks with bounded weighted in- and out-degree. Panel (a) shows the linear relationship between $\bar{\sigma }(G)$ and ${n}_{c}$, when the matrix $A$ is fixed (hence, also $n$ and $r(A)$ remain fixed). Specifically, we let $n=100$, consider a randomly generated regular graph with in- and out-degrees equal to $20$, and construct the network matrix $A$ by randomly associating a weight between 0 and 1 with each edge. Then, we stabilize the matrix $A$ by adding suitable negative constants to the diagonal weights. For each ${n}_{c}=1,\ldots ,n$, we select 50 random choices of ${n}_{c}$ control nodes. Each plotted point represents a single realization of $\bar{\sigma }(G)$. Panels (b,c) describe the tradeoff expressed by (4) when ${n}_{c}$ is fixed and equal to 20. In panel (b), for $n\in \{20,40,60,100\}$, we randonly construct 100 network matrices with edge weights between 0 and 1 and in- and out-degrees equal to 6 (each matrix is stabilized by adding negative constants to the diagonal weights). Finally, panel (c) shows two cases where either $r(A)$ or $\bar{\sigma }(G)$ remains constant (red dots in (c) and red dashed line in (b), and blue dots in (c) and blue dashed line in (b)), as *n* increases from 20 to 100.

The main result of this paper is to prove that networks that are responsive tend to be fragile. Besides its theoretical value in the fields of network science and control, our result constitutes an explanation of why several highly-optimized networks are fragile. To characterize a trade-off between fragility and responsiveness, consider the dynamic network modeled by Eq. (1) and let 1/$r(A)$ and $\bar{\sigma }(G)$ denote its fragility and responsiveness, as defined in (2) and (3), respectively. Our first result is the following inequality:

4 $$\bar{\sigma }(G)\le \frac{{n}_{{\rm{c}}}}{n}(1+\frac{4\parallel A-{A}^{{\rm{\top }}}\parallel }{3\pi }\frac{1}{r(A)})\frac{1}{r(A)},$$

where $n$ is the total number of nodes in the network and ${n}_{{\rm{c}}}$ is the number of driver nodes. The bound in Eq. (4) reveals a number of tradeoffs between the responsiveness of a network, its fragility, and the number of driver nodes. First, the fewer the driver nodes, the smaller the average singular value $\bar{\sigma }(G)$ and, consequently, the less responsive the network (see Fig. 3(a)). Second, the term $\parallel A-{A}^{\top }\parallel$ is a measure of the asymmetry of the network matrix $A$, and hence of its non-normality degree^26^. Such a measure, which reduces to zero for networks with symmetric matrices, quantifies the transient amplification of the network dynamics when subject to external stimuli^26^, and plays a fundamental role in determining the controllability degree of a network^27–30^. Assuming that $\parallel A-{A}^{\top }\parallel$ remains bounded, the larger the stability radius $r(A)$, the smaller the average singular value $\bar{\sigma }(G)$, thus proving that robust networks with linear dynamics cannot be responsive (based on the adopted definitions of robustness and responsiveness and in an asymptotic sense; see Fig. 3(b)). Third, when the network dimension $n$ grows and the number of driver nodes ${n}_{{\rm{c}}}$ remains constant, then, assuming that $\parallel A-{A}^{\top }\parallel$ remains bounded (see below), either the network becomes less responsive ($\bar{\sigma }(G)$ decreases) or more fragile ($r(A)$ decreases) (see Fig. 3(b,c)). The latter tradeoff between responsiveness and fragility as the network size increases becomes evident (i) when the network matrix *A* is symmetric, in which case, (4) simplifies to

5 $$\bar{\sigma }(G)\le \frac{{n}_{{\rm{c}}}}{n}\frac{1}{r(A)},$$

and (ii) for networks with bounded weighted in- and out-degrees (see Fig. 3).

In the case when the network matrix *A* is diagonalizable, we have the following second inequality

6 $$\bar{\sigma }(G)\le \frac{{n}_{{\rm{c}}}}{n}\frac{{\kappa }^{2}(V)}{2s(A)},$$

where $\kappa (V)$ denotes the 2-norm condition number of the eigenvector matrix $V$ (that is, $\parallel V\parallel \,\parallel {V}^{-1}\parallel$, where $V$ is an invertible matrix such that ${V}^{-1}AV$ is a diagonal matrix^31^), and

$$s(A)=\mathop{-{\rm{\max }}}\limits_{i=1,\ldots ,n}\,\Re ({\lambda }_{i}(A)),$$

measures the distance of the eigenvalues of $A$ from the imaginary axis, where $\Re (\cdot )$ denotes the real part of a complex number.

Further, it can be shown that

7 $$\frac{s(A)}{\kappa (V)}\le r(A)\le s(A).$$

Together, Eqs. (6) and (7) explain how different algebraic and geometric properties of the linear dynamic network (1) influence its responsiveness versus fragility trade-off. In particular, increasing the stability margin $s(A)$ tends to reduce the responsiveness of a network (small $\bar{\sigma }(G)$), while potentially reducing its fragility (see (7)). Similarly, highly non-normal networks (large $\kappa (V)$) can be more responsive (see (6)), yet potentially more fragile (see (7)). Thus, Eqs. (6) and (7) explain how the spectrum of a network, which determines $s(A)$, and its geometric structure, which determines $\kappa (V)$, differently affect fragility and responsiveness. In fact, since $s(A)$ describes the distance of the eigenvalues from the instability region, a small value of $s(A)$ implies that a small change of the network parameters may relocate eigenvalues that are close to the imaginary axis to the right-half complex plane (instability region). The relevance of the non-normality degree of a network on its fragility is due to its influence on the sensitivity of the eigenvalues of *A* to variations of the edge weights. Indeed, when the network is highly non-normal (large $\kappa (V)$), a small change of the edge weights may induce a significant change on the location of the eigenvalues, thus inducing dynamic instabilities even when the network eigenvalues are located far from the instability region^26^. Clearly, a network can be fragile due to either of the two causes (small $s(A)$ and large $\kappa (V)$), or to a combination of the two.

Symmetric networks, and more generally networks with *normal* adjacency matrices (a network is normal if $A{A}^{\top }={A}^{\top }A$), satisfy $\kappa (V)=1$. For these networks, $s(A)=r(A)$, and the expression in Eq. (6) reduces to

8 $$\bar{\sigma }(G)\le \frac{{n}_{{\rm{c}}}}{n}\frac{1}{2r(A)},$$

which improves upon inequality (5).

### Fragility and responsiveness in synthetic and real networks

In this section we validate the theoretical bounds presented in the previous section on synthetic and real network systems.

In Fig. 3 we illustrate the fragility versus responsiveness tradeoff for a class of randomly generated networks in which both the in- and out-degrees are bounded. This property ensures that the term $\parallel A-{A}^{\top }\parallel$ in (4) remains bounded: in fact, bounded in- and out-degrees imply that $\parallel A{\parallel }_{1}$ and $\parallel {A}^{\top }{\parallel }_{1}$ are also bounded, and so is^31^ $\Vert A-{A}^{\top }\Vert \le 2\Vert A\Vert \le 2\sqrt{\parallel A{\parallel }_{1}\,\parallel {A}^{\top }{\parallel }_{1}}$. This figure shows the tradeoff between $\bar{\sigma }(G)$ and $r(A)$, for fixed value of ${n}_{{\rm{c}}}$ and different values of $n$. It can be seen that, when $n$ grows, either $\bar{\sigma }(G)$ decreases or $r(A)$ decreases, confirming the prediction of the theoretical bound in (4).

**Figure 4:**
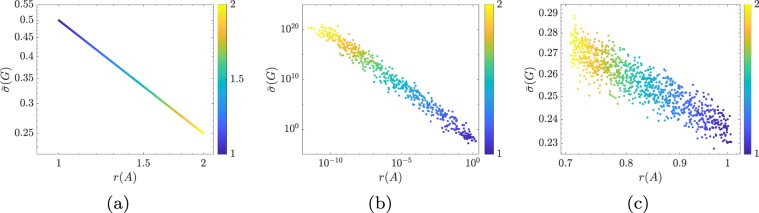
Panel (a) shows, in a logarithmic scale, the fragility versus responsiveness tradeoff for networks with matrices ${A}_{\alpha }={A}_{{\rm{skew}}}-\alpha I$, with ${A}_{{\rm{skew}}}=-\,{A}_{{\rm{skew}}}^{\top }\in {{\mathbb{R}}}^{40\times 40}$ and $1\le \alpha \le 2$. Panel (b) shows, in a logarithmic scale, the fragility versus responsiveness tradeoff for networks with matrices ${A}_{\beta }={D}_{\beta }^{-1}{A}_{{\rm{sym}}}{D}_{\beta }$, with ${A}_{{\rm{sym}}}={A}_{{\rm{sym}}}^{\top }\in {{\mathbb{R}}}^{40\times 40}$ and $1\le \beta \le 2$. Panel (c) shows the fragility versus responsiveness tradeoff for networks with matrices ${A}_{\gamma }=A+B{K}_{\gamma }$, where $A\in {{\mathbb{R}}}^{40\times 40}$ is a randomly-generated symmetric matrix, $1\le \gamma \le 2$, $B=I$, and ${K}_{\gamma }$ solves the infinite-horizon Linear Quadratic Regulator^32^ problem with matrices *Q* = *I* and $R=\gamma I$. The values of $\alpha$, $\beta$, and $\gamma$ are color coded. It should be noted that (i) fragility (1/$r(A)$) and responsiveness ($\bar{\sigma }(G)$) are directly related, and (ii) the value of $\alpha$ (stability margin), $\beta$ (non-normality degree) and $\gamma$ (control cost) determine fragility and responsiveness. In all figures we use ${n}_{{\rm{c}}}=n$ control nodes.

In Fig. 4(a,b) we illustrate the fragility versus responsiveness tradeoff for synthetic networks with matrices ${A}_{\alpha }$ and ${A}_{\beta }$, respectively, as a function of the parameters $\alpha \in {{\mathbb{R}}}_{ > 0}$ and $\beta \in {{\mathbb{R}}}_{ > 1}$. In particular, the network matrices are defined as ${A}_{\alpha }={A}_{{\rm{skew}}}-\alpha I$, where ${A}_{{\rm{skew}}}$ is a randomly generated matrix satisfying ${A}_{{\rm{skew}}}^{\top }=-\,{A}_{{\rm{skew}}}$, and ${A}_{\beta }={D}_{\beta }^{-1}{A}_{{\rm{sym}}}{D}_{\beta }$, where ${A}_{{\rm{sym}}}$ is a randomly generated stable symmetric matrix, and

$${D}_{\beta }=[\begin{array}{ccccc}1 & 0 & 0 & \cdots  & 0\\ 0 & \beta  & 0 & \cdots  & 0\\ 0 & 0 & {\beta }^{2} & \cdots  & 0\\ \vdots  & \vdots  & \vdots  & \ddots  & \vdots \\ 0 & \cdots  & \cdots  & \cdots  & {\beta }^{n-1}\end{array}].$$

It can be shown that ${A}_{\alpha }$ is always normal and stable, and that $s({A}_{\alpha })=\alpha$. Instead, ${A}_{\beta }$ is stable but non-normal, and its non-normality degree grows with $\beta$. Thus, the matrices ${A}_{\alpha }$ and ${A}_{\beta }$ allow us to isolate the different effects of the stability margin $s(A)$ and the condition number $\kappa (V)$ on fragility and responsiveness, as described in Eq. (6). Figure 4 shows how fragility and responsiveness are proportionally related, and how this relation is modulated by the parameters $\alpha$ and $\beta$. Similarly, Fig. 4(c) shows that the fragility versus responsiveness tradeoff also holds when the network matrix is $A+B{K}_{\gamma }$, where ${K}_{\gamma }$ is the optimal stabilizing controller that solves the Linear Quadratic Regulator^32^ problem with matrices *Q* = *I* and $R=\gamma I$.

**Figure 5:**
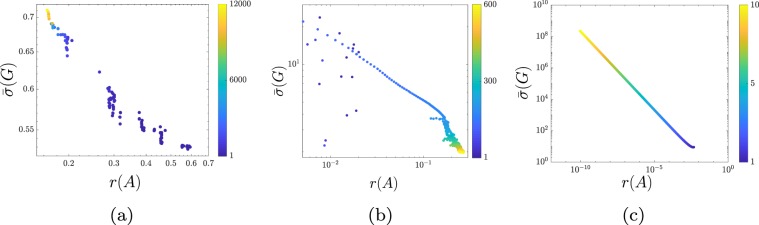
This figure shows the fragility versus responsiveness tradeoff for networks modeling ecological (a), neuronal (b), and traffic (c) systems, respectively. As predicted by our analysis, in all cases fragility (1/$r(A)$) and responsiveness ($\bar{\sigma }(G)$) are directly related, and this tradeoff is regulated by relevant network parameters (color coded; see Supplementary Information for a detailed description of these networks). Panel (a) analyzes  ecological networks obtained through the iterative Community-level Optimization algorithm applied to an ecological model^33^. Each network has $n=40$ nodes, and is obtained at different iterations of the algorithm (color coded). Panel (b) analyzes  neuronal networks with $n=100$ nodes generated by an iterative algorithm^34^ (algorithm iterations are color coded). Finally, panel (c) analyzes networks corresponding to the linearization of the dynamics of a platoon of $n=101$ vehicles (one leader, 100 followers, different velocities are color coded).

Finally, we consider three classes of networks arising in the study of ecological, neuronal, and traffic systems. In Fig. 5(a) we show the fragility vs responsiveness tradeoff for a set of ecological networks generated by a community-level optimization algorithm^33^, which iteratively modifies the trophic interactions between species to increase the total abundance of the species. We observe that, as the algorithm progresses, the generated networks become more responsive, thus leading to larger concentrations of the species, yet more fragile. In Fig. 5(b), we consider a set of neuronal networks obtained through an optimization algorithm^34^ that generates networks capable of modeling effective transmission of information between neurons. Such algorithm starts from an unstable (infinitively responsive) neuronal network, and aims to obtain a network with increased robustness while maintaining an elevated level of responsiveness. As can be seen from the figure, as the algorithm progresses, the generated networks tend to be more robust yet less responsive. Lastly, in Fig. 5(c) we consider networks arising from the linearization of the nonlinear dynamics describing platoons of vehicles at equilibrium, where all vehicles are equally spaced and travel with constant velocity $\alpha$ imposed by the leader vehicle (see Supplementary Information for more details). Depending on the velocity $\alpha$, these linearized networks feature different fragility and responsiveness properties. In particular, Fig. 5(c) shows that fragility and responsiveness are proportionally related, and that they both increase as $\alpha$ grows. This finding is consistent with the empirical evidence that perturbations to the motions of platoons moving with higher velocities are more likely to generate larger traffic waves^8^.

## Discussion

In this paper we show that an inherent tradeoff exists in linear dynamic networks, which forces efficient and highly optimized networks to be fragile to perturbation of the edge weights. In particular, we measure responsiveness of a network through the eigenvalues of the controllability Gramian, which describe how external stimuli are propagated through the network, and quantify the ability of a network to be manipulated from benign controls, to adapt in response to external excitation, and to easily transmit signals across different network locations. Our notion of fragility, instead, is determined by the eigenvalues and eigenvectors of the network matrix, and quantifies the ability of a network to maintain a stable behavior in the face of changes in the network structure and weights. As we show in the paper, the notions of network responsiveness and fragility are rigorous and general, so as to allow for the derivation of a probably correct tradeoff between fragility and responsiveness and, at the same time, to remain applicable to a broad class of natural and man-made dynamic networks.

**Figure 6:**
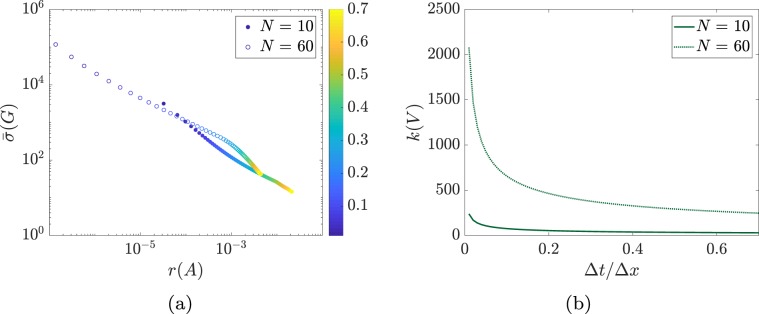
Panel (a) shows  the fragility versus responsiveness tradeoff for discrete-time networks obtained through the discretization of the partial differential equation $\frac{\partial }{\partial t}u(t,x)=\frac{\partial }{\partial x}u(t,x)$, with $x\in (\,-\,1,1)$ and $t\ge 0$. We applied a space-time discretization on a regular Δ*x*-Δ*t* grid, with $\Delta t=\delta \Delta x$ (see the Supplementary Information for further details). The value of *δ* ranges from 0.1 to 0.7, and it is color coded. This figure shows that the discussed fragility vs responsiveness tradeoff applies also to discrete-time networks. Panel (b) shows  the condition number of the network matrix, as a function of the discretization parameter *δ*. As can be seen, the smaller *δ*, the larger the non-normality and fragility degrees of the network.

Some methodological considerations are in order. First, the bounds presented in this paper, particularly Eq. (4), provide a qualitative characterization of the tradeoff between fragility and responsiveness in network systems. We remark that tighter quantitative bounds can be obtained at the expenses of a more involved technical notation by following the analysis described in the Supplementary Information. Second, our results have been derived under the assumption of linearity of the network dynamics. While this assumption is commonly and broadly used to study dynamic network systems, it is well-recognized that real-world network systems are better described by nonlinear dynamical models. Thus, our results need to be considered valid for networks with linear dynamics, or around the equilibrium points of nonlinear network processes. The development of theories and tools to study fragility and responsiveness of networks with nonlinear dynamics remains an ongoing research direction. Third and finally, although we have presented our results for networks with continuous-time dynamics, analogous results can be derived for discrete-time network systems by appropriately modifying the notions of fragility and controllability Gramian (see Supplementary Information). For instance, Fig. 6 shows the fragility vs responsiveness tradeoff for a discrete-time network obtained by discretization of the standard first-order wave equation

$$\frac{\partial }{\partial t}u(t,x)=\frac{\partial }{\partial x}u(t,x).$$

By varying the discretization parameter $\delta =\frac{\Delta t}{\Delta x}$, the obtained discretized networks feature different fragility and responsiveness properties. Figure 6(a) shows the tradeoff between network fragility and responsiveness for different values of *δ*. Similarly, Fig. 6(b) shows that the obtained discretized networks feature higher non-normality degrees as the parameter *δ* decreases.

## Methods

### Stability and fragility of network systems

The linear dynamic network in Eq. (1) is stable if, in the absence of inputs, all the state trajectories converge to the zero state, and it is unstable if there exists a state trajectory that diverges to infinity. Stability depends on the eigenvalues of the matrix $A$: precisely, the linear dynamic network (1) is stable if and only if all the eigenvalues of $A$ have negative real parts.

Linear dynamic networks often result from the small signal linearization around a desired equilibrium point of more realistic nonlinear dynamic networks. When the linearized network is unstable, arbitrarily small perturbations of the state $x(t)$ (or of the input $u(t)$) may result in increasingly large state trajectories, forcing the original nonlinear network system to depart from the desired equilibrium towards different, and possibly undesired, limiting configurations (see Fig. 2(a)). On the other hand, stability of (1) ensures a certain degree of robustness of the stable behavior against parameter variations. This degree of robustness can be quantified by the size of the smallest matrix Δ such that the perturbed matrix $A+\Delta$ is unstable. Formally, if

$$r(A):\,=\,{\rm{\min }}\,\{\parallel \Delta \parallel \,:\,\Delta \,{\rm{such}}\,{\rm{that}}\,A+\Delta \,{\rm{is}}\,{\rm{unstable}}\},$$

denotes the stability radius of *A*, then the network is *fragile* when $r(A)$ is small, and it is robust otherwise. Figure 2(c) shows the behavior of a robust network, where a small variation of the network matrix preserves stability so that the equilibrium configuration exhibits only a small change after the perturbation. Instead, Fig. 2(d) shows the behavior of a fragile network, where a small perturbation destabilizes the linearized network and induces a large variation in the equilibrium configuration of the original nonlinear network. This behavior is typical of large ecological systems^4^, as illustrated numerically in Fig. 1.

### Responsiveness and controllability of networks

Responsiveness describes the ability to influence the state of a network by acting on the inputs. This property can be made more precise by specifying three cases, namely the degree of controllability, the effect of noisy inputs, and the size of the response to impulsive inputs. In all of these cases the notion of controllability Gramian plays an essential role. The controllability Gramian is defined as

9 $$G:={\int }_{0}^{\infty }\,{e}^{At}B{B}^{\top }{e}^{{A}^{\top }t}dt.$$

A network is controllable if it is possible to drive its state from the zero equilibrium state to any final target state ${x}_{f}$ by a suitable choice of the input signal $u(t)$. It is well known that a network is controllable if and only if the Gramian is invertible^32^. Moreover, the minimum energy of the input to drive the state to ${x}_{f}$ is given by ${x}_{f}^{\top }{G}^{-1}{x}_{f}$ (see Supplementary Information). From these arguments we can argue that a network is difficult to control when such energy is large, that is, when *G*^−1^ is “large”. Because of the inequality $\bar{\sigma }(G)\ge n$/${\rm{tr}}({G}^{-1})$ (see Supplementary Information), a network is difficult to control if $\bar{\sigma }(G)$ is small.

If, instead, $u(t)$ in Eq. (1) is white noise with covariance matrix equal to the identity matrix, then $x(t)$ is a stationary random process whose covariance matrix coincides with the Gramian^35^. In this context the Gramian quantifies the amplification of the noise that results from the network dynamics. Depending on the different situations, a large amplification can yield either negative or positive effects. On the one hand, it could produce undesirable fluctuations, as for instance high volatility in certain economic networks^36,37^ or congestions in traffic dynamics^38^. On the other hand, large fluctuations allow a network to explore the state space in search for more efficient configurations in both natural and in artificial optimization processes. For instance noise plays a crucial role in stochastic optimization algorithms, including randomized search, evolutionary algorithms, simulated annealing and genetic algorithms^39^. In decision making, noise is of critical importance to find the right balance between the random exploration phase, which allows to learn the environment, and the exploitation phase, in which the best choice is taken based on the available information^40^, [Chapter 10]. Furthermore, models of natural evolution based on a similar paradigm have been proposed (see the models based on optimization over fitness, evolutionary, or adaptive lanscapes^41,42^).

Finally the Gramian can be used to describe the size of the response of a network in reaction to impulsive inputs, where a large Gramian implies the existence of high energy responses. This property has been used, for instance, to characterize the ability of a neuronal network to produce large transients dynamics for the generation of complex movements^34,43^, and to measure the ability of a neuronal network to facilitate short term memory by increasing persistence of neuronal activity^44–46^.

The above discussion motivates the advantages of having responsive dynamic networks. Different functions of the singular values ${\sigma }_{i}(G)$ of the Gramian *G* can be used to quantify the responsiveness of a network (see Supplementary Information). In this paper we use the average singular value

$$\bar{\sigma }(G)=\frac{1}{n}\,\mathop{\sum }\limits_{i=1}^{n}\,{\sigma }_{i}(G)$$

as the metric for the network responsiveness, so that responsive networks are characterized by large values of $\bar{\sigma }(G)$.

## Supplementary information

Supplementary Information.

## References

[1] Bassett DS, Bullmore E (2006). Small-world brain networks. The neuroscientist.

[2] Pilosof S, Porter MA, Pascual M, Kéfi S (2017). The multilayer nature of ecological networks. Nature Ecology & Evolution.

[3] Motter AE, Myers SA, Anghel M, Nishikawa T (2013). Spontaneous synchrony in power-grid networks. Nature Physics.

[4] Allesina S, Tang S (2015). The stability-complexity relationship at age 40: a random matrix perspective. Population Ecology.

[5] Sritharan D, Sarma SV (2014). Fragility in dynamic networks: application to neural networks in the epileptic cortex. Neural computation.

[6] McCulloch M, Falter J, Trotter J, Montagna P (2012). Coral resilience to ocean acidification and global warming through ph up-regulation. Nature Climate Change.

[7] Kinney R, Crucitti P, Albert R, Latora V (2005). Modeling cascading failures in the north american power grid. The European Physical Journal B-Condensed Matter and Complex Systems.

[8] Bando M, Hasebe K, Nakayama A, Shibata A, Sugiyama Y (1995). Dynamical model of traffic congestion and numerical simulation. Physical Review E.

[9] Newman, M. E. J. *Networks: An Introduction* (Oxford University Press, 2010).

[10] Watts DJ, Strogatz SH (1998). Collective dynamics of ‘small-world’ networks. Nature.

[11] Barabási AL, Albert R (1999). Emergence of scaling in random networks. Science.

[12] Fortunato S (2010). Community detection in graphs. Physics Reports.

[13] Boccaletti S, Latora V, Moreno Y, Chavez M, Hwang DU (2006). Complex networks: Structure and dynamics. Physics Reports.

[14] Liu YY, Slotine JJ, Barabási AL (2011). Controllability of complex networks. Nature.

[15] Pasqualetti F, Zampieri S, Bullo F (2014). Controllability metrics, limitations and algorithms for complex networks. IEEE Transactions on Control of Network Systems.

[16] Gu, S. *et al*. Controllability of structural brain networks. *Nature Communications* **6** (2015). 10.1038/ncomms9414 PMC4600713 26423222

[17] Yan G (2015). Spectrum of controlling and observing complex networks. Nature Physics.

[18] Skardal PS, Arenas A (2015). Control of coupled oscillator networks with application to microgrid technologies. Science Advances.

[19] Hinrichsen D, Pritchard AJ (1986). Stability radii of linear systems. Systems & Control Letters.

[20] Qiu L (1995). A formula for computation of the real stability radius. Automatica.

[21] Kailath, T. *Linear Systems* (Prentice-Hall, 1980).

[22] Lynn CW, Bassett DS (2019). The physics of brain network structure, function and control. Nature Reviews Physics.

[23] Stacey, W. *et al*. Emerging roles of network analysis for epilepsy. *Epilepsy Research* 106255 (2019). 10.1016/j.eplepsyres.2019.106255 PMC6990460 31855828

[24] Liu, J. *et al*. Analysis and control of a continuous-time bi-virus model. *IEEE Transactions on Automatic Control*, In press (2019).

[25] Betzel RF, Gu S, Medaglia JD, Pasqualetti F, Bassett DS (2016). Optimally controlling the human connectome: the role of network topology. Scientific Reports.

[26] Trefethen, L. N. & Embree, M. *Spectra and Pseudospectra: the Behavior of Nonnormal Matrices and Operators* (Princeton University Press, 2005).

[27] Pasqualetti, F. & Zampieri, S. On the controllability of isotropic and anisotropic networks. In *IEEE Conf*. *on Decision and Control*, 607–612 (Los Angeles, CA, USA, 2014).

[28] Zhao S, Pasqualetti F (2019). Networks with diagonal controllability gramians: Analysis, graphical conditions, and design algorithms. Automatica.

[29] Baggio, G. & Zampieri, S. On the relation between non-normality and diameter in linear dynamical networks. In *European Control Conference*, 1839–1844 (Limassol, Cyprus, 2018).

[30] Olshevsky A (2016). Eigenvalue clustering, control energy, and logarithmic capacity. Systems & Control Letters.

[31] Meyer, C. D. *Matrix Analysis and Applied Linear Algebra* (SIAM, 2001).

[32] Hespanha, J. P. *Linear Systems Theory* (Princeton University Press, 2009).

[33] Suweis S, Simini F, Banavar JR, Maritan A (2013). Emergence of structural and dynamical properties of ecological mutualistic networks. Nature.

[34] Hennequin G, Vogels TP, Gerstner W (2014). Optimal control of transient dynamics in balanced networks supports generation of complex movements. Neuron.

[35] Caines PE (1988). Linear stochastic systems.

[36] Acemoglu D, Carvalho VM, Ozdaglar A, Tahbaz-Salehi A (2012). The network origins of aggregate fluctuations. Econometrica.

[37] Huang, Q., Yuan, Y., Goncalves, J. & Dahleh, M. A. *h*_2_ norm based network volatility measures. In *American Control Conference*, 3310–3315 (Portland, OR, 2014).

[38] Hoogendoorn SP, Bovy PH (2001). State-of-the-art of vehicular traffic flow modelling. Proceedings of the Institution of Mechanical Engineers, Part I: Journal of Systems and Control Engineering.

[39] Spall, J. C. *Introduction to Stochastic Search and Optimization: Estimation*, *Simulation*, *and Control* (John Wiley & Sons, 2003).

[40] Powell, W. B. *Approximate Dynamic Programming: Solving the Curses of Dimensionality* (John Wiley and Sons, 2007).

[41] Kauffman, S. A. *The Origins of Order: Self-Organization and Selection in Evolution* (Oxford University Press, 1993).

[42] Svensson, E. & Calsbeek, R. *The Adaptive Landscape in Evolutionary Biology* (Oxford University Press, 2012).

[43] Hennequin G, Vogels TP, Gerstner W (2012). Non-normal amplification in random balanced neuronal networks. Phys. Rev. E.

[44] Ganguli S, Huh D, Sompolinsky H (2008). Memory traces in dynamical systems. Proceedings of the National Academy of Sciences.

[45] Ganguli S, Latham P (2009). Feedforward to the past: The relation between neuronal connectivity, amplification, and short-term memory. Neuron.

[46] Goldman MS (2009). Memory without feedback in a neural network. Neuron.

[47] Bascompte J, Jordano P, Olesen JM (2006). Asymmetric coevolutionary networks facilitate biodiversity maintenance. Science.

