# Supplementary material for: Fragility Limits Performance in Complex Networks
Source: Sci Rep. 2020 Feb 4;10:1774. doi: 10.1038/s41598-020-58440-6 (PMC7000764; doi:10.1038/s41598-020-58440-6)
Supplement: Supplementary file 1 — Supplementary Information. [file 41598_2020_58440_MOESM1_ESM.pdf]

Supplementary Information
Fragility Limits Performance in Complex Networks

Fabio Pasqualetti[1], Shiyu Zhao[2], Chiara Favaretto[3] & Sandro Zampieri[*,3]

[1] *Department of Mechanical Engineering, University of California, Riverside*
[2] *Department of Electrical and Computer Engineering, Westlake University, Hangzhou, China*
[3] *Department of Information Engineering, University of Padova, Padova*
[*] *To whom correspondence should be addressed (email: sandro.zampieri@unipd.it).*

1 Network controllability, stability, and fragility

1.1 The controllability Gramian and its properties

Consider a dynamic network with graph $\mathcal{G}$ and dynamics

$$\frac{d}{dt}x(t) = Ax(t) + Bu(t), \quad t \geq 0, \tag{1}$$

where $x(t) \in \mathbb{R}^n$ and $u(t) \in \mathbb{R}^{n_c}$ denote the network state and input, respectively. The matrix $A \in \mathbb{R}^{n\times n}$ denotes a weighted adjacency matrix of $\mathcal{G}$, while $B \in \mathbb{R}^{n\times n_c}$ denotes the input matrix. The network is stable when the eigenvalues of A have negative real part, in which case the state x vanishes asymptotically when the network has no input. The network (1) is said to be controllable when its controllability Gramian G_{t_f} is invertible[1, Thm 3.4.1]

$$G_{t_f} := \int_0^{t_f} e^{At}BB^\mathsf{T}e^{A^\mathsf{T}t}dt, \tag{2}$$

where $t_f > 0$ denotes the control horizon, and A^T denotes the transpose of A.

If the network (1) is controllable, then there exist control inputs to drive the network state from any initial state $x(0) = x_0$ to any final state $x(t_f) = x_f$. It is known that this happens if and only if G_{t_f} is invertible. In particular, the input with minimum energy to drive the network state from x_0 to x_f is given by

$$u_{\text{opt}}(t) = B^\mathsf{T}e^{-A^\mathsf{T}(t-t_f)}G_{t_f}^{-1}(x_f - e^{At_f}x_0), \tag{3}$$

whose energy is

$$\int_0^{t_f} u_{\text{opt}}^\mathsf{T}(t)u_{\text{opt}}(t)dt = (x_f - e^{At_f}x_0)^\mathsf{T}G_{t_f}^{-1}(x_f - e^{At_f}x_0).$$

When $t_f = \infty$ and the network is stable,

$$G := G_\infty = \int_0^\infty e^{At} B B^\mathsf{T} e^{A^\mathsf{T} t} dt, \tag{4}$$

and the energy to drive the state from x_0 to x_f equals

$$\int_0^\infty u_{\text{opt}}^\mathsf{T}(t) u_{\text{opt}}(t) dt = x_f^\mathsf{T} G^{-1} x_f \tag{5}$$

The controllability Gramian G can be computed in different ways. For instance, G is the unique solution of the Lyapunov equation[1, Thm 3.3.1]

$$AG + GA^\mathsf{T} = -BB^\mathsf{T}. \tag{6}$$

Moreover[2],

$$G = \frac{1}{2\pi \mathrm{i}} \int_\Gamma (zI - A)^{-1} (-BB^\mathsf{T}) (zI + A^\mathsf{T})^{-1} dz,$$

where Γ is a curve in the complex plane that encloses all the eigenvalues of A. By choosing Γ as the semi-circle with infinite radius enclosing the stable half plane, we obtain

$$G = \frac{1}{2\pi} \int_{-\infty}^{+\infty} (\omega \mathrm{i} I - A)^{-1} BB^\mathsf{T} (\omega \mathrm{i} I - A)^{-H} d\omega. \tag{7}$$

where $(\omega \mathrm{i} I - A)^{-H}$ denotes the inverse of the complex conjugate of $\omega \mathrm{i} I - A$. While equations (4), (7) and (6), are valid only when A is stable, the expression (2) is valid also for unstable networks.

The controllability Gramian can be used to quantify the responsiveness of a network to external stimuli. In particular, several scalar metrics can be defined to measure the "size" of the controllability Gramian, and therefore quantify the control energy needed to reach particular states. In this paper, we use $\bar{\sigma}(G) = \frac{1}{n} \sum_{i=1}^n \sigma_i(G) = \text{tr}(G)/n$ to quantify the responsiveness of a network, which is also an indirect measure of the average energy needed to control the network. Different metrics are also interesting. For instance, $\sigma_{\min}(G) = \min\{\sigma_1(G), \ldots, \sigma_n(G)\} = 1/\|G^{-1}\|$, whose inverse $1/\sigma_{\min}(G)$ quantifies the largest control energy over all possible target states. Clearly, $\sigma_{\min}(G) \le n/\text{tr}(G^{-1}) \le (\det(G))^{1/n} \le \bar{\sigma}(G) \le \sigma_{\max}(G)$, where $\text{tr}(G)$, $\det(G)$, and $\sigma_{\max}(G)$ denote the trace, determinant, and largest eigenvalue of the Gramian G. Notice that $\text{tr}(AB) \le \text{tr}(A^2)\text{tr}(B^2)$ for any positive semidefinite matrices A and B. Then, the following inequality holds:

$$n^2 = \text{tr}(I)^2 = \text{tr}(G^{1/2} G^{-1/2})^2 \le \text{tr}(G)\text{tr}(G^{-1})$$

This implies that $\text{tr}(G^{-1})/n$ grows when $\bar{\sigma}(G) = \text{tr}(G)/n$ becomes small.

1.2 The stability radius of a network and its fragility

When the network (1) is stable, the following definition of stability radius quantifies its distance to instability ($\|\cdot\|$ denotes the Euclidean norm):

$$r(A) := \min\{\|\Delta\| \;:\; \Delta \in \mathbb{C}^{n \times n}, A + \Delta \text{ is unstable}\}.$$

The index $r(A)$, namely, the stability radius of A, quantifies the degree of stability of (1), as it quantifies the minimum size of a perturbation of the weights that renders the network unstable. Conversely, $1/r(A)$ can be used to measure the degree of fragility of (1) with respect to changes of its weights. It can be shown that [3, Prop 4.1]

$$r(A) = \min_{\omega \in \mathbb{R}} \sigma_{\min}(\omega \mathrm{i} I - A) = \frac{1}{\max_{\omega \in \mathbb{R}} \|(\omega \mathrm{i} I - A)^{-1}\|}.$$

2 Network responsiveness and fragility

In this section we characterize analytical relationships between the responsiveness and fragility degrees of a network. Recall that[4,5] $\sigma_{\min}(X)\mathrm{tr}(Y) \leq \mathrm{tr}(XY) \leq \sigma_{\max}(X)\mathrm{tr}(Y)$. Then, from (4) we obtain

$$\mathrm{tr}(G) = \mathrm{tr}\left(\int_0^\infty e^{At} B B^\mathsf{T} e^{A^\mathsf{T} t} dt\right) \leq \sigma_{\max}\left(\int_0^\infty e^{A^\mathsf{T} t} e^{At} dt\right) \mathrm{tr}(BB^\mathsf{T}) = n_c \left\| \int_0^\infty e^{A^\mathsf{T} t} e^{At} dt \right\|. \quad (8)$$

Further, using (7), we have

$$\begin{aligned}
\int_0^\infty e^{A^\mathsf{T} t} e^{At} dt &= \frac{1}{2\pi} \int_{-\infty}^{+\infty} (\omega \mathrm{i} I - A^\mathsf{T})^{-1} (-\omega \mathrm{i} I - A)^{-1} d\omega \\
&= \frac{1}{2\pi} \int_{-\infty}^{+\infty} [(-\omega \mathrm{i} I - A)(\omega \mathrm{i} I - A^\mathsf{T})]^{-1} d\omega \\
&= \frac{1}{2\pi} \int_{-\infty}^{+\infty} [\omega^2 I + AA^\mathsf{T} - \omega \mathrm{i} A + \omega \mathrm{i} A^\mathsf{T}]^{-1} d\omega \\
&= \frac{1}{2\pi} \int_{-\infty}^{+\infty} [\omega^2 I + AA^\mathsf{T} + \omega \mathrm{i}(A^\mathsf{T} - A)]^{-1} d\omega.
\end{aligned}$$

We will now derive a family of upper bounds for $\bar{\sigma}(G) = \mathrm{tr}(G)/n$, which will be parametrized by the scalar $\alpha \in [0, 1]$. For the results in the main text, only the case of $\alpha = 1/2$ will be used. However, the family of upper bounds derived here remain of general and independent interest, as they provide different insights into $\bar{\sigma}(G)$ for different values of α. Let ω satisfy

$$\omega^2 I + \omega \mathrm{i}(A^\mathsf{T} - A) \geq \alpha^2 \omega^2 I$$

or, equivalently,

$$(1 - \alpha^2)\omega^2 I + \mathrm{i}(A^\mathsf{T} - A)\omega \geq 0.$$

Observe that $A^\mathsf{T} - A$ is skew symmetric. Then, $\mathrm{i}(A^\mathsf{T} - A)$ is a Hermitian matrix, and it features only real eigenvalues that are symmetric with respect to the origin. Namely, if μ is an eigenvalue of $\mathrm{i}(A^\mathsf{T} - A)$, so is $-\mu$. This implies that the maximum and the minimum eigenvalues of $i(A^\mathsf{T} - A)$ are $\|A - A^\mathsf{T}\|$ and $-\|A - A^\mathsf{T}\|$, respectively. We conclude that $(1 - \alpha^2)\omega^2 I + \mathrm{i}(A^\mathsf{T} - A)\omega \geq 0$ if and only if $|\omega| \geq \bar{\omega}$ where $\bar{\omega} := \frac{\|A - A^\mathsf{T}\|}{1 - \alpha^2}$.

Let $\int_0^\infty e^{A^\mathsf{T} t} e^{At} dt = I_1 + I_2$, where

$$I_1 := \frac{1}{2\pi} \int_{-\bar{\omega}}^{\bar{\omega}} [\omega^2 I + AA^\mathsf{T} + \omega \mathrm{i}(A^\mathsf{T} - A)]^{-1} d\omega$$

$$I_2 := \frac{1}{2\pi} \int_{-\infty}^{-\bar{\omega}} [\omega^2 I + AA^\mathsf{T} + \omega \mathrm{i}(A^\mathsf{T} - A)]^{-1} d\omega + \frac{1}{2\pi} \int_{\bar{\omega}}^{+\infty} [\omega^2 I + AA^\mathsf{T} + \omega \mathrm{i}(A^\mathsf{T} - A)]^{-1} d\omega.$$

Notice that

$$I_1 \leq \frac{\bar{\omega}}{\pi} \max_{\omega \in [0, \bar{\omega}]} \|(\omega \mathrm{i} I - A^\mathsf{T})^{-1}\|^2 \leq \frac{\bar{\omega}}{\pi} \max_{\omega \in [0, \infty]} \|(\omega i I - A)^{-1}\|^2 = \frac{\bar{\omega}}{\pi} \frac{1}{r(A)^2} = \frac{1}{\pi} \frac{\|A - A^\mathsf{T}\|}{1 - \alpha^2} \frac{1}{r(A)^2}.$$

Similarly,

$$I_2 \leq \frac{1}{2\pi} \int_{-\infty}^{-\bar{\omega}} [\alpha^2 \omega^2 I + AA^\mathsf{T}]^{-1} d\omega + \frac{1}{2\pi} \int_{\bar{\omega}}^{+\infty} [\alpha^2 \omega^2 I + AA^\mathsf{T}]^{-1} d\omega \leq \frac{1}{2\pi} \int_{-\infty}^{+\infty} [\alpha^2 \omega^2 I + AA^\mathsf{T}]^{-1} d\omega.$$

Let U be a unitary matrix satisfying $AA^\mathsf{T} = U^H \mathrm{diag}\,\{\sigma_i(A)^2\} U$, where $\sigma_i(A)$ denotes the i-th singular value of A. Then,

$$I_2 \leq \frac{1}{2\pi} U^H \int_{-\infty}^{+\infty} \mathrm{diag}\,\left\{ \frac{1}{\alpha^2 \omega^2 + \sigma_i(A)^2} \right\} d\omega U = \frac{1}{2\pi\alpha^2} U^H \mathrm{diag}\,\left\{ \left[\frac{\alpha}{\sigma_i(A)} \arctan\left(\frac{\alpha}{\sigma_i(A)} \omega \right) \right]_{-\infty}^{\infty} \right\} U$$
$$= \frac{1}{2\pi\alpha^2} U^H \mathrm{diag}\,\left\{ \frac{\alpha}{\sigma_i(A)} \pi \right\} U = \frac{1}{2\alpha} U^H \mathrm{diag}\,\left\{ \frac{1}{\sigma_i(A)} \right\} U.$$

Consequently, we have

$$\|I_2\| \leq \frac{1}{2\alpha \sigma_{\min}(A)},$$

where $\sigma_{\min}(A)$ is the minimum singular value of A. Notice now that $r(A) \leq \sigma_{\min}(A)$. Thus,

$$\|I_2\| \leq \frac{1}{2\alpha} \frac{1}{r(A)}.$$

Finally, for each value of α we obtain

$$\left\| \int_0^\infty e^{A^\mathsf{T} t} e^{At} dt \right\| \leq \frac{1}{\pi} \frac{\|A - A^\mathsf{T}\|}{1 - \alpha^2} \frac{1}{r(A)^2} + \frac{1}{2\alpha} \frac{1}{r(A)},$$

and, for $\alpha = 1/2$,

$$\left\| \int_0^\infty e^{A^\mathsf{T} t} e^{At} dt \right\| \leq \left(1 + \frac{4\|A - A^\mathsf{T}\|}{3\pi} \frac{1}{r(A)} \right) \frac{1}{r(A)} \tag{9}$$

Substituting (9) into equation (8) yields

$$\bar{\sigma}(G) \leq \frac{n_c}{n} \left(1 + \frac{4\|A - A^\mathsf{T}\|}{3\pi} \frac{1}{r(A)} \right) \frac{1}{r(A)}.$$

If A is symmetric, then it is more convenient to choose $\alpha = 1$, which yields

$$\bar{\sigma}(G) \leq \frac{n_c}{n} \frac{1}{2r(A)}.$$

2.1 The role of the non-normality degree of A

In this section we assume that the matrix A is diagonalizable, and characterize the role of the non-normality degree of A with respect to its fragility and responsiveness. Observe that

$$\begin{aligned}\left\|\int_0^\infty e^{A^\mathsf{T}t}e^{At}dt\right\| &= \left\|\int_0^\infty V^{-H}e^{\Lambda^* t}V^H V e^{\Lambda t}V^{-1}dt\right\| = \|V\|^2\|V^{-1}\|^2\left\|\int_0^\infty e^{\Lambda^* t}e^{\Lambda t}dt\right\| \\ &= \kappa^2(V)\left\|\int_0^\infty e^{2\Re(\Lambda)t}dt\right\| = \kappa^2(V)\max_i \frac{1}{-2\Re(\lambda_i(A))} = \frac{\kappa^2(V)}{2s(A)},\end{aligned}$$

where $s(A) = -\max_i \Re(\lambda_i(A)) > 0$. Substituting the above result into (8) yields

$$\bar{\sigma}(G) \leq \frac{n_c}{n}\frac{\kappa^2(V)}{2s(A)}.$$

Observe that $s(A)$ represents the distance of the eigenvalues of A from the instability region. On the other hand, $\kappa(V)$ is instead related the sensitivity of the eigenvalues of A to possible perturbations[13]. Thus, both the distance of the eigenvalues of A from the imaginary axis as well as their sensitivity to perturbations contribute to the fragility degree of a network.

3 Numerical studies

3.1 Ecological networks

An ecological dynamical network is described by the following set of differential equations [6]:

$$\frac{d}{dt}x_i(t) = c_i x_i(t) + x_i(t)\sum_{j=1}^n M_{ij}x_j(t) \qquad i = 1,\dots,n \tag{10}$$

where n denotes the number of species, $x_i(t)$ is the density of the species i, and c_i and M_{ij} are network parameters that regulate the interaction rates among the species. The network (10) can be written in vector form as

$$\frac{d}{dt}x(t) = \text{diag}(x(t))(c + Mx(t)) = f(x(t)), \tag{11}$$

where $\text{diag}(x)$ is the diagonal matrix defined by the species vector x, c is the vector of c_i, and M is the matrix of the coefficients M_{ij}. Let $x^* \in \mathbb{R}^n_+$ an equilibrium point of (11). Then, either $x^* = 0$, which corresponds to the case where all species are extinct, or x^* solves the equations $c = -Mx^*$. The stability of an equilibrium point x^* can be assessed through the linearized system

$$\frac{d}{dt}\delta x(t) = A\delta x(t), \tag{12}$$

where $A = \text{diag}(x^*)M$ is the Jacobian matrix of (11) at the point x^*.

An ecological network is called *mutualistic* if the species can be divided into two classes, where the species of each class benefit from the species in the other class. In a mutualistic network, the matrix M can be partitioned as

$$M = \begin{bmatrix} M_{PP} & M_{PA} \\ M_{AP} & M_{AA} \end{bmatrix}, \tag{13}$$

where the matrices M_{PP} and M_{AA} have non-positive entries, while the matrices M_{PA} and M_{AP} have non-negative entries. In Figure 1 in the main text we consider a three-dimensional network of two species of plants x_1 and x_2 and one species of animals x_3. Figures 1(a) and 1(b) highlight the difference between the dynamics of a stable and an unstable equilibrium: in both cases the three states are at equilibrium until time $t = 10$, when they are slightly perturbed by a vector ε, with $\|\varepsilon\| = 0.1$. Figures 1(c) and 1(d), instead, highlight the difference between a robust and a fragile system. The state is at equilibrium until time $t = 10$, when a slight variation of the parameters changes M into $M + \Delta$, with $\|\Delta\| = 0.01$. The parameters used to obtain Figure 1 are below:

Parameters of Figure 1(a)

$$M = \begin{bmatrix} -0.9144 & 0 & 0.5726 \\ 0 & -0.5291 & 0.2423 \\ 0.2673 & 0.4296 & -0.8914 \end{bmatrix}, c = \begin{bmatrix} 0.1925 & 0.6696 & 2.0752 \end{bmatrix}^{\mathsf{T}},$$

$$x^* = \begin{bmatrix} 3.3885 & 3.5897 & 5.0745 \end{bmatrix}^{\mathsf{T}}, \varepsilon = \begin{bmatrix} 0.0015 & 0.0886 & 0.0463 \end{bmatrix}^{\mathsf{T}}.$$

Parameters of Figure 1(b)

$$M = \begin{bmatrix} 0.5006 & 0 & 0.8294 \\ 0 & -0.8598 & 0.1686 \\ 0.1167 & 0.0605 & -0.5828 \end{bmatrix}, c = \begin{bmatrix} -3.1616 & 0.7901 & 1.5232 \end{bmatrix}^{\mathsf{T}},$$

$$x^* = \begin{bmatrix} 1.2955 & 1.5130 & 3.0300 \end{bmatrix}^{\mathsf{T}}, \varepsilon = \begin{bmatrix} 0.0764 & 0.0572 & 0.0300 \end{bmatrix}^{\mathsf{T}}.$$

Parameters of Figure 1(c)

$$M = \begin{bmatrix} -0.8420 & 0 & 0.1789 \\ 0 & -0.9837 & 0.1776 \\ 0.7760 & 0.0756 & -0.3488 \end{bmatrix}, c = \begin{bmatrix} 0.5612 & 2.1414 & 2.9214 \end{bmatrix}^{\mathsf{T}},$$

$$x^* = \begin{bmatrix} 5.1633 & 5.9979 & 21.1595 \end{bmatrix}^{\mathsf{T}}, \Delta = 10^{-3} \times \begin{bmatrix} 1.0 & 2.0 & 6.6 \\ 3.8 & 1.9 & 4.8 \\ 2.0 & 4.0 & 2.1 \end{bmatrix}.$$

Parameters of Figure 1(d)

$$M = \begin{bmatrix} 0.5338 & 0 & 0.1369 \\ 0 & -0.6678 & 0.0040 \\ 0.7117 & 0.0414 & -0.7027 \end{bmatrix}, c = \begin{bmatrix} -3.5456 & 2.7163 & 3.0247 \end{bmatrix}^{\mathsf{T}},$$

$$x^* = \begin{bmatrix}4.3473 & 4.1209 & 8.9499\end{bmatrix}^\mathsf{T}, \Delta = 10^{-3} \times \begin{bmatrix}1.4 & 4.7 & 4.7\\ 1.1 & 4.7 & 3.1\\ 0.92 & 3.3 & 3.2\end{bmatrix}.$$

Figure 2 in the main text shows the percentage of stable equilibra in ecological networks of growing dimension. For each dimension $n \in \{5, \dots, 320\}$, we generate 500 Watts-Strogatz (WS) small-world networks $\mathcal{G}_n$ with mean degree $2\lfloor n/6 \rfloor$, and rewiring probability 0.5. The weighted matrix M is such that $M_{ij} = 0$ if (i, j) is not an edge of G_n, and, otherwise, it equals a random number uniformly distributed in $[0, 1]$. The diagonal entries of M are randomly selected from the uniform distribution in $[-n, 0]$. The equilibrium vector x^* is formed by randomly selecting its entries from the uniform distribution in $[0, 5]$, and by letting $c := -Mx^*$. Finally, stability of x^* is assessed by computing the eigenvalues of the Jacobian matrix $A = \text{diag}(x^*)M$.

Figure 5(a) shows the trade-off between the stability radius and the average singular value for the linearization of a sequence of mutualistic ecological networks generated by the algorithm proposed in [7]. This algorithm iteratively modifies the network weights so as to increase the total abundance of the species. Specifically, the algorithm starts by letting $x_i^{*(0)} = 1$, for each $i = 1, \dots, n$ and by taking a random matrix $M^{(0)}$, which respects the sign constraints of a mutualistic network. The initial vector $c^{(0)}$ is consequently determined as $c^{(0)} = -M^{(0)} x^{*(0)}$. In each step of the procedure, $c^{(k)}$ is fixed and equal to $c^{(0)}$. At each step, two weights $M_{ij}^{(k)} \neq 0$ and $M_{rs}^{(k)} = 0$ (with $M_{ij}^{(k)}$ and $M_{rs}^{(k)}$ being entries of $M_{PA}^{(k)}$ or $M_{AP}^{(k)}$) are randomly selected and switched, so that $M_{ij}^{(k+1)} = M_{rs}^{(k)}$ and $M_{rs}^{(k+1)} = M_{ij}^{(k)}$. If the sum of the entries of the new equilibrium point $x^{*(k+1)}$ is smaller than or equal to the sum of the entries of the previous equilibrium point $x^{*(k)}$, then the swap is discarded and $M^{(k+1)} = M^{(k)}$. Otherwise the swap is accepted, and $M^{(k+1)}$ is updated accordingly. At each step, $A^{(k)}$ denotes the linearization matrix of the mutualistic ecological network associated with $M^{(k)}$ and $c^{(k)}$, and $G^{(k)}$ denotes the associated Gramian with $B = I$. The coordinates of the points in the plot correspond to $r(A^{(k)})$ and $\bar{\sigma}(G^{(k)})$.

3.2 Neuronal networks

Following[8] a network of neurons can be modelled by the differential equation

$$\tau \frac{d}{dt} x(t) = -x(t) + Mx(t) + e(t), \tag{14}$$

where $x(t)$ is the vector of spiking rates of the neurons, $e(t)$ is the column vector with the external inputs, τ is the time constant of the neurons, and the matrix M describes the strength of connections among neurons. Because each neuron can be either excitatory or inhibitory, then the matrix M obeys Dale's law, namely, its columns are either non-negative or non-positive. This implies that $x(t)$ and M can be partitioned as follows

$$x(t) = \begin{bmatrix} x_E(t) \\ x_I(t) \end{bmatrix}, \quad M = \begin{bmatrix} M_{EE} & -M_{EI} \\ M_{IE} & -M_{II} \end{bmatrix}, \tag{15}$$

where $x_E(t)$ and $x_I(t)$ contain the states of the excitatory and inhibitory neurons, respectively, and the matrices M_{EE}, M_{EI}, M_{IE} and M_{II} are non-negative.

We follow the algorithm in[9] to construct a sequence of matrices M that obey Dale's law and tend to minimize the value of s such that $\int_0^\infty e^{(M-sI)^\mathsf{T} t} e^{(M-sI)t} dt = \frac{1}{\epsilon}$ (ϵ-smoothed spectral abscissa[10]). We refer interested reader to [9] for a detailed description of this algorithm. To generate Figure 5 (b), we consider a network of dimension $n = 100$ and $n_E = n_I = 50$. Let $M^{(k)}$ be the coupling matrix at the k-th iteration of the algorithm in[9], and let $A^{(k)} = (M^{(k)} - I)/\tau$. We then compute the controllability Gramian $G^{(k)}$ with $B = I$. Figure 5 (b) in the main text shows the relationship between the stability radius $r(A^{(k)})$ and the average singular $\bar{\sigma}(G^{(k)})$.

3.3 Traffic Networks

Following[11], a traffic network where vehicles drive as an aligned platoon is described by the equations

$$\begin{aligned} \frac{d}{dt} p_i(t) &= v_i(t), \\ \frac{d}{dt} v_i(t) &= f_i(p_i(t), p_{i+1}(t), v_i(t), u_i(t)) = a\left[\tanh\left(p_{i+1} - p_i\right) - v_i\right] + u_i, \end{aligned} \tag{16}$$

where p_i and v_i are the position and the velocity of the i-th vehicle $i \in \{1, \dots, n-1\}$, respectively, $\tanh$ is the hyperbolic tangent function, and u_i is an external input. We assume that the n-th vehicle plays the role of leader, whose velocity is constant and equal to α, and whose position enters as external input to the system (16). When all vehicles also move with velocity α, the system (16) read as

$$\frac{d}{dt} \bar{p}_i(t) = \alpha, \qquad i = 1, \dots, n-1 \tag{17a}$$

$$\frac{d}{dt} \bar{v}_i(t) = a\left[\tanh\left(\bar{p}_{i+1}(0) - \bar{p}_i(0)\right) - \alpha\right] = 0, \qquad i = 1, \dots, n-1, \tag{17b}$$

whose solution is

$$\bar{p}_i(t) = \alpha t + \bar{p}_i(0), \qquad \bar{p}_{i+1}(0) - \bar{p}_i(0) = \tanh(\alpha)^{-1}, \qquad i = 1, \dots, n-1 \tag{18a}$$

$$\bar{v}_i(t) = \alpha, \qquad i = 1, \dots, n-1. \tag{18b}$$

In order to analyze the dynamics of system (16) in the neighborhood of the particular trajectory (18), we linearize the nonlinear system (16) around the trajectory (18). Let us define $\delta_i(t) = p_i(t) - \bar{p}_i(t)$, and consider $x = \left[\delta_1,\ \frac{d}{dt}\delta_1, \dots, \delta_{n-1},\ \frac{d}{dt}\delta_{n-1}\right]^\mathsf{T}$ and $u = [u_1,\ \cdots,\ u_{n-1},\ \delta_n]^\mathsf{T}$ as the state and input vectors of the linearized system. Then,

$$\frac{d}{dt} x = Ax + Bu,$$

where the matrices $A \in \mathbb{R}^{2(n-1)\times 2(n-1)}$ and $B \in \mathbb{R}^{2(n-1)\times n}$ are defined as

$$A = \begin{bmatrix} 0 & 1 & 0 & 0 & 0 & \cdots & 0 & 0 \\ -ab & a & ab & 0 & 0 & \cdots & 0 & 0 \\ 0 & 0 & 0 & 1 & 0 & \cdots & 0 & 0 \\ 0 & 0 & -ab & a & ab & \cdots & 0 & 0 \\ \vdots & \vdots & \vdots & \ddots & \ddots & \ddots & \vdots & \vdots \\ \vdots & \vdots & \vdots & \vdots & \ddots & \ddots & \ddots & \vdots \\ 0 & 0 & 0 & 0 & 0 & \ddots & 0 & 1 \\ 0 & 0 & 0 & 0 & 0 & \cdots & -ab & a \end{bmatrix}, \; B = \begin{bmatrix} 0 & 0 & \cdots & 0 & 0 \\ 1 & 0 & \cdots & 0 & 0 \\ 0 & 0 & \cdots & 0 & 0 \\ 0 & 1 & \cdots & 0 & 0 \\ \vdots & \vdots & & \vdots & \vdots \\ \vdots & \vdots & & \vdots & \vdots \\ 0 & 0 & \cdots & 0 & 0 \\ 0 & 0 & \cdots & 1 & ab \end{bmatrix}, \text{ with } b = 1 - \alpha^2.$$

Figure 5(c) in the main text has been generated by considering a linearized system of dimension 200, derived by 101 vehicles (one leader, and 100 followers), 100 control nodes, driven by the 101 inputs $(u_1, \ldots, u_{100}, \delta_n)$, which is determined by the selected constant velocity α of the leader. Different values of α are color coded in the figure.

3.4 Networks obtained from discretization of the wave equation

Consider the first-order wave equation[12, Ch. VII]

$$\frac{\partial}{\partial t} w(t, z) = \frac{\partial}{\partial z} w(t, z), \tag{19}$$

with $z \in (-1,\, 1)$ and boundary values $w(1, t) = 0$ for all $t \geq 0$. We discretize (19) using a regular grid and a centered difference scheme for the spatial coordinate. This yields,

$$\frac{\partial}{\partial z} w(t, -1 + i\Delta z) \approx \frac{w(t, -1 + (i+1)\Delta z) - w(t, -1 + (i-1)\Delta z)}{2\Delta z},$$

and Eq. (19) becomes

$$\frac{d}{dt} w(t, -1 + i\Delta z) \approx \frac{w(t, -1 + (i+1)\Delta z) - w(t, -1 + (i-1)\Delta z)}{2\Delta z}, \tag{20}$$

with $i \in \{1, \ldots, N\}$, where the number of grid points N determines the discretization step $\Delta z = 2/N$. In vector form, the system of discretized equations (20), read as

$$\frac{d}{dt} w = \frac{1}{2\Delta z} D w,$$

where $D \in \mathbb{R}^{(N-1)\times(N-1)}$ with

$$D = \begin{bmatrix} -1 & 1 & & 0 \\ -1 & 0 & \ddots & \\ & \ddots & \ddots & 1 \\ 0 & & -1 & 0 \end{bmatrix},$$

and where we have used $w(t, 1) = w(t, -1 + N\Delta z) = 0$ and $w(t, -1) = w(t, -1 + \Delta z)$ at all times. We then discretize the temporal coordinate using the third-order Adams-Bashforth formula and obtain

$$v(k+1) \approx v(k) + \frac{\Delta t}{12}\frac{1}{2\Delta z}D\left(23v(k) - 16v(k-1) + 5v(k-2)\right),$$

where $v(k) = w(k\Delta t) \in \mathbb{R}^{N-1}$. Finally, letting

$$x(k) = \begin{bmatrix} v(k+2) \\ v(k+1) \\ v(k) \end{bmatrix},$$

we obtain

$$x(k+1) = Ax(k), \tag{21}$$

where $\delta = \frac{\Delta t}{\Delta x}$ and

$$A = \begin{bmatrix} I_{N-1} & 0_{N-1} & 0_{N-1} \\ I_{N-1} & 0_{N-1} & 0_{N-1} \\ 0_{N-1} & I_{N-1} & 0_{N-1} \end{bmatrix} + \frac{\delta}{2}\begin{bmatrix} \frac{23}{12}D & -\frac{16}{12}D & \frac{5}{12}D \\ 0_{N-1} & 0_{N-1} & 0_{N-1} \\ 0_{N-1} & 0_{N-1} & 0_{N-1} \end{bmatrix}.$$

Finally, we add a control input and use the following equations

$$x(k+1) = Ax(k) + Bu(k), \qquad \text{with} \qquad B = \begin{bmatrix} I_{N-1} \\ 0_{N-1} \\ 0_{N-1} \end{bmatrix}, \tag{22}$$

to evaluate the network controllability Gramian, and its eigenvalues as a function of the parameters N and δ. Figure 6(a) in the main text shows the fragility versus responsiveness tradeoff for the discrete-time network (22) for the value of δ that ranges from 0.1 to 0.7. Figure 6(b) shows the condition number of the network matrix, as a function of δ. It can be seen, the smaller δ, the larger the non-normality and fragility degrees of the network.

Supplementary References

1. B. M. Chen, Z. Lin, and Y. Shamash, "Linear Systems Theory: A Structural Decomposition Approach", *Birkhäuser*, 2004.

2. P. Lancaster, "Explicit solutions of linear matrix equations", *Siam review*, vol. 12, no. 4, pp. 544–566, 1970.

3. D. Hinrichsen and A. J. Pritchard, "Real and complex stability radii: a survey", *Control of uncertain systems*. Springer, 1990, pp. 119–162.

4. S.-D. Wang, T.-S. Kuo, and C.-F. Hsu, "Trace bounds on the solution of the algebraic matrix riccati and lyapunov equation", *IEEE Transactions on Automatic Control*, vol. 31, no. 7, pp. 654–656, 1986.

5. Y. Fang, K. A. Loparo, and X. Feng, "Inequalities for the trace of matrix product", *IEEE Transactions on Automatic Control*, vol. 39, no. 12, pp. 2489–2490, 1994.

6. S. Allesina and S. Tang, "The stability–complexity relationship at age 40: a random matrix perspective", *Population Ecology*, vol. 57, no. 1, pp. 63–75, 2015.

7. S. Suweis, F. Simini, J. R. Banavar, and A. Maritan, "Emergence of structural and dynamical properties of ecological mutualistic networks", *Nature*, vol. 500, no. 7463, pp. 449–452, 2013.

8. G. Hennequin, T. P. Vogels, and W. Gerstner, "Non-normal amplification in random balanced neuronal networks", *Phys. Rev. E*, vol. 86, p. 011909, 2012.

9. ——, "Optimal control of transient dynamics in balanced networks supports generation of complex movements", *Neuron*, vol. 82, no. 6, pp. 1394 – 1406, 2014.

10. J. Vanbiervliet, B. Vandereycken, W. Michiels, S. Vandewalle, and M. Diehl, "The smoothed spectral abscissa for robust stability optimization," *SIAM Journal on Optimization*, vol. 20, no. 1, pp. 156–171, 2009.

11. M. Bando, K. Hasebe, A. Nakayama, A. Shibata, and Y. Sugiyama, "Dynamical model of traffic congestion and numerical simulation", *Physical review E*, vol. 51, no. 2, p. 1035, 1995.

12. L. N. Trefethen and M. Embree, "Spectra and pseudospectra: the behavior of nonnormal matrices and operators", *Princeton University Press*, 2005.

13. M. Asllani, R. Lambiotte and T. Carletti, "Structure and dynamical behavior of non-normal networks", *Science advances*, vol. 4, no. 12, pp. eaau9403, 2018.
